# Serological Evidence of Antibodies to Rift Valley Fever Virus in Wild and Domestic Animals in Bauchi State, Nigeria

**DOI:** 10.1155/2022/6559193

**Published:** 2022-03-16

**Authors:** Y. J. Atuman, C. A. Kudi, P. A. Abdu, O. O. Okubanjo, Y. Wungak, H. G. Ularamu, A. Abubakar

**Affiliations:** ^1^National Veterinary Research Institute Vom Outstation Laboratory, Bauchi, Nigeria; ^2^Department of Veterinary Medicine, Faculty of Veterinary Medicine, Ahmadu Bello University, Zaria, Nigeria; ^3^Department of Veterinary Parasitology and Entomology, Ahmadu Bello University, Zaria, Nigeria; ^4^Viral Research Division, National Veterinary Research Institute, Vom, Nigeria; ^5^Force Animal Branch Department, Nigeria Police Force Headquarters, Abuja, Nigeria

## Abstract

Rift Valley fever (RVF) is an arthropod-borne zoonotic disease responsible for severe outbreaks in livestock and humans with concomitant economic losses in many countries in sub-Saharan Africa. The study, therefore, investigated the seroprevalence of the Rift Valley fever virus (RVFV) among wild and domestic animals. Blood samples were collected between 2013 and 2015 from 106 wild animals, 300 cattle (*Bos indicus*), and 200 horses (*Equus caballus*), respectively, in Yankari Game Reserve (YGR) and Sumu Wildlife Park (SWP) in Bauchi state, Nigeria. Harvested sera from blood were evaluated for the presence of anti-RVFV IgM/IgG antibodies. The overall seroprevalence in cattle was 11.3% (*p* = 0.677; 95% CI: 0.624–0.730) and in wildlife was 8.5% (*p* = 0.006; 95% CI: 0.00–0.60). The diversity of wildlife species sampled indicated seropositivity of 36.0% in waterbuck (*Kobus ellipsiprymus*), 25.0% in elephant (*Loxodonta africana*), 12.5% in eland (*Taurotragus oryx*), and 8.3% in wildebeest (*Connochaetes taurinus*). Whereas, samples from zebra (*Equus quagga crawshayi*), kudu (*Tragelaphus strepsiceros*), and hartebeest (*Alcelaphus buselaphus caama*) did not show detectable antibodies to RVFV, and seroprevalence in female (15.0%) wildlife species was higher than in males (4.5%) (*p* = 0.061). Classification of cattle into breed and sex showed no significant difference in seropositivity. Seropositivity of 12.0% was observed in White Fulani, 12.1% in Red Bororo, and 7.8% in Sokoto Gudali breeds of cattle (*p* = 0.677). Whereas, seropositivity of 13.6% was observed in females and 6.4% observed in males (*p* = 0.068). This study indicated the presence of antibodies to RVFV among some wild animals and cattle in the absence of a reported outbreak in the study area. The circulation of RVFV in the study area may pose a significant health risk to livestock, wildlife, and humans. Therefore, surveillance for RVFV should be intensified targeting mosquito vectors and humans in Bauchi state, Nigeria.

## 1. Introduction

Rift Valley fever (RVF) is an emerging zoonotic disease of public and animal health concern which is endemic in many countries in sub-Saharan Africa [1]. The Rift Valley fever virus (RVFV) is an arbovirus of the genus *Phlebovirus* of the family Phenuiviridae and replicates in mosquitoes and domestic and wild animals [2]. The disease is associated with severe outbreaks in livestock and is characterized by a sudden onset of abortions and high neonatal mortality resulting in significant economic losses [1]. The virus is transmitted between wildlife and domestic animals and humans via arthropod vectors [3]. While in the eggs of the *Aedes* mosquitoes, the virus can persist through droughts [4]. These mosquitoes can acquire RVFV from feeding on infected animals [5] and are capable of transmitting the virus directly to their offspring via eggs leading to a new generation of infected mosquitoes [6, 7]. Humans are at great risk of infection through direct contact with blood, body fluids, or tissues of infected animals, mainly livestock such as cattle, sheep, goats, and camels and wildlife or through infected mosquito bites [8–10]. The disease in humans is characterized by self-limiting febrile illness, and only 1-2% of cases result in severe complications [10–13]. Rift Valley fever virus spreads by the movement of infected hosts or vectors [9, 14], and since the discovery of RVFV [15], many RVF outbreaks and continuous detection of serological positive individuals (animals or humans) were reported in sub-Saharan Africa, Madagascar, Arabian Peninsula, and the Middle East with major impacts on human and animal health [16–19]. In West Africa, outbreaks of RVF in animals and humans have recently been reported in Niger and Mali with high numbers of deaths among humans and animals [20–22].

In Nigeria, the virus was first isolated in a sheep in 1959 [23]. Virological and serological surveys in the country revealed the presence of antibodies to RVFV in livestock including sheep, goats, cattle, horses, and camels [24–29] and also in humans [10, 11, 30, 31]. A recent study in Nigeria has demonstrated increase in the mosquito population, water bodies, and vegetation as crucial drivers of RVF in cattle herds in Niger state [32]. A similar study among livestock and wildlife species with such ecological determinants is essential for designing an effective control program. The presence of antibodies to RVFV in wildlife species has been documented in many African countries [33–36]. However, in Nigeria, little work has been done to demonstrate the presence of antibodies to RVFV in wildlife over the last decades [24], indicating that this is a neglected area of research. Similar studies will provide insight on the role of wild animals' in RVFV maintenance and potential spillover to domestic animals. Domestic livestock sometimes do share the same range with wildlife in Yankari Game Reserve (YGR) and Sumu Wildlife Park (SWP) in Bauchi state, Nigeria, and there is concern that wildlife may form a reservoir niche for RVFV; consequently, there is a need for concise epidemiological information on the presence and distribution of antibodies to RVFV in these species of wildlife and domestic animals to aid in the design and implementation of disease control programs. The study determined the seroprevalence of RVFV in some wildlife species and livestock in YGR and SWP in Bauchi state, Nigeria.

## 2. Materials and Methods

### 2.1. Study Area

**Figure 1:**
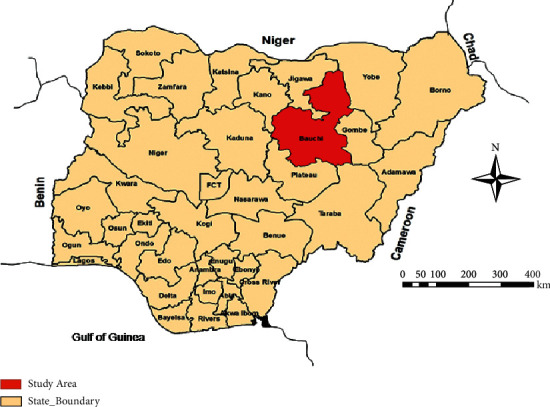
Map of Nigeria showing Bauchi state. Source: modified from the administrative map of Nigeria (http://www.theodora.com/maps) (https://doi.org/10.1155/2020/3642793).

**Figure 2:**
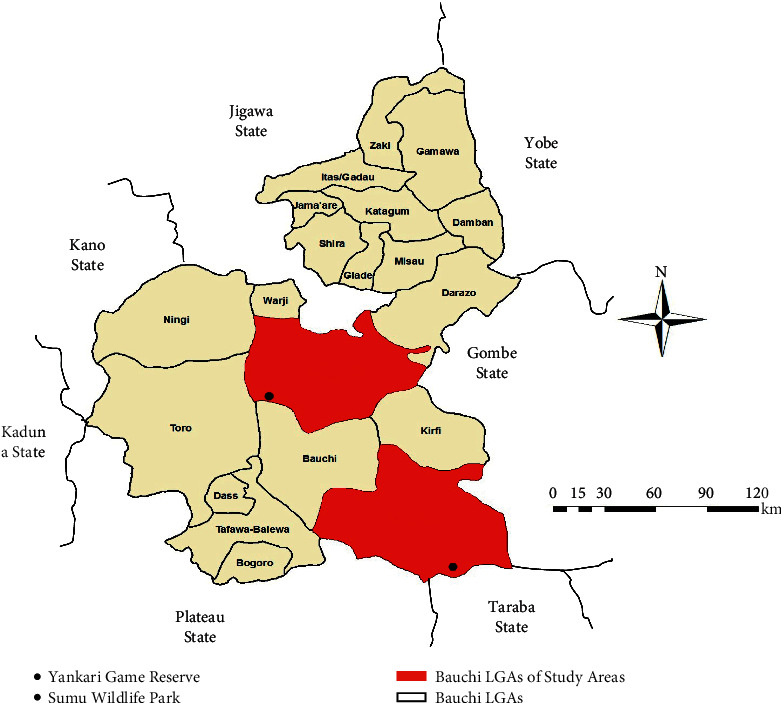
Map of Bauchi state showing study areas. Source: modified from the administrative map of Nigeria (http://www.theodora.com/maps) (https://doi.org/10.1155/2020/3642793). LGA, local government area.

The study was conducted in Bauchi state (Figure 1), located between longitude 9° 15ˈE to 10° to 43ˈE and latitude 9° 55ˈN to 12° 45ˈN in the northern Guinea/Sudan savannah zone of Nigeria. It has an annual rainfall of between 875 and 1075 mm. The study locations were Yankari Game Reserve (YGR) and Sumu Wildlife Park (SWP) of Bauchi state and the settlements surrounding them (Figure 2). The study was approved by the Animal Ethics Committee of the Ahmadu Bello University, Zaria, Nigeria (ref ABUCAUC/2018/012). The YGR is located in the south-central part of Bauchi state. The reserve has an area of about 2,244 square kilometres [37]. It lies between latitude 9°34′ and 10°00′ N and longitude 10°17′ and 10°47′E, and it has characteristic savannah vegetation, including swamps with river floodplains, water surfaces, and grassland and thick bushes [37]. It harbors about 50 species of mammals and over 350 species of birds and is one of the few remaining areas where wild animals are protected in their natural habitat in Nigeria [37]. The SWP is located 60 km north of the state capital Bauchi (Figure 2). It harbors wildlife species including impala (*Aepyceros melampus*), eland (*Taurotragus oryx*), zebra (*Equus quagga crawshayi*) kudu (*Tragelaphus strepsiceros*), blue wildebeest (*Connochaetes taurinus*), and giraffe (*Giraffa camelopardalis*) [38].

### 2.2. Study Design and Target Animals

We carried out a cross-sectional study between 2013 and 2015. The target animals were wildlife species within Yankari Game Reserve (YGR) which included elephant (*Loxodonta africana*), waterbuck (*Kobus ellipsiprymus*), and hartebeest (*Alcelaphus buselaphus caama*) from YGR and those from Sumu Wildlife Park (SWP) including eland (*Taurotragus oryx*), kudu (*Tragelaphus strepsiceros*), and blue wildebeest (*Connochaetes taurinus*). Simple random sampling was used to identify herds of cattle and or households with horses to be sampled. Any herd with 10 cattle and above or a household with at least two horses living at the fringes of YGR and SWP was selected for sampling. Also, horses used for patrol purposes by rangers within SWP were sampled. The sample size was determined using the following formula: *n* = *Z*^2^ pq/d^2^ [39], where, *n* represents the minimum sample size, Z represents the appropriate value for the normal standard deviation set 95% CI or 1.96, p represents the expected prevalence, and *q* represents the complementary probability. Expected prevalence was set at 50% with 4% absolute precision, and a 95% confidence interval was used. We arrived at 600, but eventually collected 606 samples from 106 wildlife species (waterbuck 11, elephant 4, hartebeest 1, eland 24, wildebeest 12, kudu 1, and zebra 53), 300 cattle, and 200 horses.

### 2.3. Sample Collection

Wildlife species were immobilized for sample collection using a combination of etorphine hydrochloride (M99^**®**^ Krüger-Med, South Africa) at 0.5–2 mg/kg and azaperone (Stresnil^**®**^, Janssen Pharmaceuticals (Pty.) Ltd., South Africa). It was administered IM from the ground using a Dan-Inject® rifle (Dan-Inject APS, Sellerup Skovvej, Denmark). Cattle and horses were profiled individually for sex and breed documentation and were restrained properly by their owners for sample collection. A volume of 10 mL of blood was collected from the jugular vein of each animal and dispensed into plain vacutainer bottles. All samples were transported in a cold box with ice to the National Veterinary Research Institute Laboratory, Bauchi. Serum samples were harvested from blood into 2 mL cryovials after spinning for 10 min at 1200 g and were divided into aliquots, labelled, and kept at −20°C until used.

### 2.4. Rift Valley Fever Virus Antibody Detection by Enzyme-Linked Immunosorbent Assay

To determine for past exposure of RVF virus infection, serum samples from domestic and wild animals were evaluated using the ID screen^**®**^ RVF competition multispecies ELISA kits (ID-Vet Innovative Diagnostics, Montpellier, France). The cELISA is based on recombinant nucleoprotein and detects anti-RVF virus IgG antibody. Briefly, 50 ul each of ELISA buffer and test sera were added to each well containing the RVFV antigen-coated test plates. Positive and negative control sera were added to the designated wells of each test plate and were incubated for 45 min at 37°C. The plates were emptied and washed three times with 300 ul of wash solutions, and 100 ml of the diluted conjugate was added to all the plates and incubated for 30 min at 21°C. The plates were then emptied and washed 3 times with 300 ul washing solution, and 100 ul of the substrate solution was dispensed to all the wells and were incubated for 15 min at 21°C in the dark. Thereafter, 100 ul of stop solution was added to all the wells. Readings were taken using a spectrophotometer Multiskan^**®**^ ELISA reader (Thermo Scientific, USA), and the optical density (OD) was determined at 450 nm. The positivity of each sample was determined by calculating its competition percentage (CP). Samples with CP = ≤40% were considered positive for RVFV antibodies and those with CP = >50% were declared negative for RVFV antibodies.

### 2.5. Statistical Analysis

Data were analysed using GraphPad Prism version 7. Results obtained were expressed as percentages and levels of association between seropositivity and sex, breed, age, and animal species and were obtained using the chi-square test. Values of *p* < 0.05 were regarded as statistically significant.

## 3. Results

Overall apparent seroprevalence of RVFV in domestic and wild animals was 7.1% with cattle having seroprevalence of 11.3%, wildlife 8.5%, and none of the sera from horses show detectable antibodies to RVFV (Tables 1 and 2). Also, of the seven wildlife species tested, antibodies to RVFV were detected in waterbuck (10.4%) wildebeest (11.3%), eland (22.6%), and elephant (3.8%) (*p* < 0.05), and none of the sera from zebra, kudu, and hartebeest show detectable antibodies to RVFV (Table 1). In wildlife, a seroprevalence of 15.0% was observed in females and 4.5% in males (*p* > 0.05) (Table 1). There was no significant difference between seropositivity among breeds of cattle (*p* > 0.05). However, seroprevalences of 12.1%, 12.0%, and 7.8% were observed in Red Bororo, White Fulani, and Sokoto Gudali breeds, respectively, whereas in males and females, a seroprevalence of 6.38% and 13.6%, respectively (*p* > 0.05) (Table 2), were observed.

## 4. Discussion

To date, published information on the occurrence of RVFV in wild and domestic animals is limited in Nigeria [24], and wildlife, livestock, and humans are competing for spaces and available resources in the YGR and SWP leading to porous frontiers and potential for transmission of zoonoses where infected animals pose a risk of infection to susceptible in-contact animals and humans. This statement is supported by other workers, who reported that RVFV can easily be transmitted at the wildlife-livestock-human interface [40–42]. Given the fact that pasteurization of milk is most often not adequately done and consumption of raw milk is a common practice among the nomadic pastoralists in Nigeria [43], this may also lead to human infection with RVFV from infected cattle. An association between consumption of unprocessed milk and human infection with RVFV has been reported during RVF outbreaks in South Africa [3]. Although no clinical cases were reported during the study period, the presence of antibodies in the absence of clinical cases and/or outbreaks have previously been reported in Nigeria [29, 32]. Whereas in sub-Saharan Africa, it was reported in cattle, goats, and buffaloes in Mozambique [13, 44] among cattle and goats in South Africa [45] and sheep and goats in Kenya [46] and Uganda [47]. The result from this study complements other reports in Bauchi state, Nigeria, where clinical cases of febrile illnesses (haemorrhagic fever syndromes) and associated deaths are likely occurring among animals and humans but unreported and in some instances linked to malaria and/or Lassa fever [48], raising concerns about the capacity for alertness and preparedness for the detection of RVFV activity among livestock and humans in the area. Thus, the circulation of RVFV in the study area may be a significant cause of both human and animal illnesses, and hence, RVF should be included among the differential diagnoses for cases presented with haemorrhagic fever syndromes.

The presence of RVFV antibodies in waterbuck, elephant, eland, and wildebeest is not surprising as there has been evidence of exposure in similar species in other countries [35, 49–51]. It is indicative that wildlife plays an important role in the epidemiology of RVF in the study area as a maintenance host for the spread to mosquitos and humans. Further studies on the duration and the extent of RVFV viremia in wildlife could therefore clarify the potential role of wildlife in maintaining the disease in the environment. From the results of this study, one can assume that both zebra and horses which had no detectable RVFV antibodies may not support RVFV replication and spread, and similar findings were observed during studies on RVFV antibodies in Kenyan wildlife [35]. A survey of RVFV infection in susceptible hosts is an important tool for risk assessment [46]. Therefore, data generated on species susceptibility and on animal demographics can provide useful information in identifying RVF risk areas to develop an effective surveillance system to curtail potential outbreaks in livestock and humans in the country.

The fact that waterbucks showed high titers of antibodies and live next to water ways makes them more prone to be maintenance hosts of the RVF virus. Furthermore, YGR has characteristic savannah vegetation, including swamps with river floodplains and water surfaces which could support the proliferation of *Aedes* and *Culex* mosquitoes' vectors for easy replication and transmission of the RVF virus in the habitat. It has been shown that the RVF virus can persist in an environment as long as the virus remains in a mosquito breeding site once introduced [47]. The ecological factors in YGR may probably have contributed to the currently observed seroprevalence in the waterbuck and other animals in the study area. A similar study on cattle herds around the national park in Borgu, Niger state, Nigeria, with an ecological habitat of high mosquito population, water bodies, and thick vegetation cover demonstrated high RVFV seropositivity in the cattle [32].

The possible explanation for the presence of RVFV in Bauchi state, Nigeria, is that both Nigeria and its neighboring countries fall within the normal endemic area of RVFV, and there is uncontrolled movement of livestock across international and state borders with interactions of domestic animals and wildlife at grazing and water points in YGR and SWP in Bauchi state [38]. Studies have shown that both mixing of wildlife with cattle herds and increasing cattle herd size were associated with a higher seroprevalence of RVFV [40, 45, 52, 53]. This finding is consistent with the reported outbreaks of RVF in ruminants in the Niger Republic, which were associated with animal movements within and from neighboring countries and likely contacts with infected vectors [21, 22].

The higher RVFV seroprevalence in females compared to male cattle observed during this study has also been previously reported for cattle [11]. This could be due to the high preference for female cattle by nomadic pastoralists who keep them for a longer period for reproductive purposes, thereby, increasing their risk of exposure to RVFV. Rift Valley fever virus-specific antibodies were detected in all the breeds of cattle examined during the study. This is indicative of natural infection as RVF vaccination has never been practiced in Nigeria [29], and susceptibility of livestock to RVFV has been reported irrespective of breed of the animals [26]. The results indicate exposure of both male and female wild and domestic animals to RVFV, raising concerns about the possibility that these species may pose a significant threat to wildlife conservation and health risk for livestock and human hosts. Control measures should be geared toward creating awareness among the general public on the zoonotic implications of RVF. Further studies are needed to isolate and characterize RVF circulating virus in wild and domestic animals from the study area. Also, since there is increasing potential for RVFV spillover at the wildlife-livestock-human interface through vector transmission and direct contact with infected blood and tissues in the study area, it is recommended that vector ecology, veterinary investigation of abortions, and activities linked with wildlife hunting and bushmeat consumption be prioritized during RVFV surveillance activities.

## 5. Conclusion

This study has found RVFV seroprevalence of 11.3% in cattle and 8.5% in wildlife in Bauchi state, Nigeria, whereas horses and zebra did not show any detectable antibodies to RVFV. Eventhough samples from wildlife were few compared to that of cattle and horses due to the challenges associated with blood sampling in wildlife species, the study provided insight into the potential for the sylvatic occurrence of RVFV in Nigeria and would serve as a baseline for further studies. There is a need for both active and passive surveillance of RVFV in sentinel species such as sheep and goats, wildlife, mosquito vectors, and humans in the study area. The need for public health intervention by the government and other stakeholders in Nigeria to improve public health awareness against this zoonotic disease cannot be overemphasized.

## Figures and Tables

**Table 1:** Seroprevalence of Rift Valley fever virus in wildlife from Yankari Game Reserve and Sumu Wildlife Park in Bauchi state, Nigeria.

Variable	Number sampled (%)	Number positive (%)	*P* value	Confidence interval at 95%
YGR				
Waterbuck	11 (10.4)	4 (36.4)		
Elephant	4 (3.8)	1 (25.0)		
Hartebeest	1 (0.9)	0 (0.0)		

SWP				
Eland	24 (22.6)	3 (12.5)		
Wildebeest	12 (11.3)	1 (8.3)		
Kudu	1 (0.9)	0 (0.0)		
Zebra	53 (50.0)	0 (0.0)		
Overall	106 (100.0)	9 (8.5)	0.006	0.00–0.60

Sex				
Male	66 (62.3)	3 (4.5)		
Female	40 (37.7)	6 (15.0)		
Overall	106 (100.0)	9 (8.5)	0.061	0.063–1.147

**Table 2:** Seroprevalence of Rift Valley fever virus in cattle around Yankari Game Reserve and Sumu Wildlife Park in Bauchi state, Nigeria.

Variable	Number sampled (%)	Number positive (%)	*P* value	Confidence interval at 95%
Breed				
Red Bororo	58 (19.3)	7 (12.1)		
Sokoto Gudali	51 (17.0)	4 (7.8)		
White Fulani	191 (63.7)	23 (12.0)		
Overall	300 (100.0)	34 (11.3)	0.677	0.624–0.730

Sex				
Male	94 (31.3)	6 (6.4)		
Female	206 (68.7)	28 (13.6)		
Overall	300 (100.0)	34 (11.3)	0.068	0.173–1.085

## Data Availability

The data used to support the findings of this study are included within the article and are available from the corresponding author upon request.
